# Thermogravimetric/Thermal–Mass Spectroscopy Insight into Oxidation Propensity of Various Mechanochemically Made Kesterite Cu_2_ZnSnS_4_ Nanopowders

**DOI:** 10.3390/ma17061232

**Published:** 2024-03-07

**Authors:** Katarzyna Lejda, Janusz Partyka, Jerzy F. Janik

**Affiliations:** 1 Faculty of Energy and Fuels, AGH University, al. Mickiewicza 30, 30 059 Kraków, Poland; 2 Faculty of Materials Science and Ceramics, AGH University, al. Mickiewicza 30, 30 059 Kraków, Poland

**Keywords:** kesterite semiconductor, mechanochemical synthesis, kesterite oxidation susceptibility, kesterite thermal stability, TGA/DTA-QMS analysis

## Abstract

Thermogravimetry coupled with thermal analysis and quadrupole mass spectroscopy TGA/DTA-QMS were primarily used to assess the oxidation susceptibility of a pool of nanocrystalline powders of the semiconductor kesterite Cu_2_ZnSnS_4_ for prospective photovoltaic applications, which were prepared via the mechanochemically assisted synthesis route from two different precursor systems. Each system, as confirmed by XRD patterns, yielded first the cubic polytype of kesterite with defunct semiconductor properties, which, after thermal annealing at 500 °C under neutral gas atmosphere, was converted to the tetragonal semiconductor polytype. The TGA/DTA-QMS determinations up to 1000 °C were carried out under a neutral argon Ar atmosphere and under a dry, oxygen-containing gas mixture of O_2_:Ar = 1:4 (vol.). The mass spectroscopy data confirmed that under each of the gas atmospheres, a distinctly different, multistep evolution of such oxygen-bearing gaseous compounds as sulfur oxides SO_2_/SO_3_, carbon dioxide CO_2_, and water vapor H_2_O was taking place. The TGA/DTA changes in correlation with the nature of evolving gases helped in the elucidation of the plausible chemistry linked to kesterite oxidation, both in the stage of nanopowder synthesis/storage at ambient air conditions and during forced oxidation up to 1000 °C in the dry, oxygen-containing gas mixture.

## 1. Introduction 

The kesterite Cu_2_ZnSnS_4_ semiconductor is widely considered to be a readily accessible and environment-friendly candidate for making new generations of photovoltaic cells [1,2,3]. Chemically, it is a complex quaternary metal sulfide that poses significant problems both with its stoichiometry and its structural coherence in the synthesis stage. The chemical nature and plausible structure disorder also suggest that the compound may be susceptible to oxidation in an ambient humid air starting from the synthesis stage, going through storage/manipulations, and ending with viable applications. Indeed, our recent studies focused on this aspect, support such predictions for a range of kesterite-type nanopowders, with the conclusion that all manipulations of kesterite have to be carried out so as to minimize its exposure to air [4,5]. As for potential applications, it has yet to be proven that suitable engineering of a layered structure of a kesterite-based PV cell would prevent the cell from chemical deterioration in humid air even at ambient temperature conditions. In this regard, the “wet” mechanochemical synthesis method that we used, relying on high-energy ball milling of the precursors slurried in xylene, appears upfront to favor low oxygen contents in the raw products since the reactions are confined to a closed volume of the air-tight grinding bowl. However, the final preparation step of recovering the raw product by the evaporation of xylene in air, as is routinely performed, leads to quite prolonged contact with air of the freshly made high-surface-area nanopowder, which favors some materials’ oxidation. As described by us earlier, the as-prepared nanopowders are made of the cubic kesterite polytype (tentatively called prekesterite) that does not show semiconductor properties. A follow-up annealing of the prekesterite at 500 °C under a neutral gas atmosphere results in the formation of the tetragonal polytype of the kesterite semiconductor as exemplified by the work-up of nanopowders from the mechanochemical synthesis of the compound from various precursor systems mastered by us [6,7,8].

One of the primary oxygen sources in kesterite preparation is adventitious amounts of oxygen in the solid substrates, such as the elemental metals and metal sulfide powders. The preparation work-up and, specifically, the drying stage in air in the “wet” mechanochemical synthesis method, constitutes yet another oxygen source, possibly even more acute, since one deals at this stage with very reactive, high-surface-area nanopowders exposed to air for relatively long and variable periods of time. As a result, such oxidation by-products are detected for kesterite samples as metal sulfates including copper sulfate pentahydrate CuSO_2_•5H_2_O, zinc sulfate monohydrate ZnSO_4_•H_2_O, and, possibly, hydrated tin oxide SnO_2_•xH_2_O not only in the mechanochemical synthesis method [4,5,9] but also in other preparation routes [10,11,12]. It is instructive to note that in some kesterite studies, the presence of oxygen-bearing species is evident from inspection of the data, although it is not specifically acknowledged or appropriately commented on by authors [13] (e.g., XPS survey scans with the apparent O 1s band in the range of 529–531 eV for oxides or the S2p_3/2_/S2p_1/2_ bands in the range of 167–172 eV for the sulfate group [SO_4_]^−2^).

Thermogravimetry/thermal analysis (TGA/DTA) is typically carried out under rising temperature conditions at a rate of a few to several degrees per minute and spanning a wide temperature range of heating samples from ambient to a level of several hundred degrees or more. By coupling with mass spectroscopy, e.g., quadrupole mass spectroscopy, QMS, for the detection of off-gases, the combined TGA/DTA-QMS method enables correlation of the mass changes with heat effects and the type of evolved gaseous compounds due to thermal decomposition. In this regard, kesterite is known to be thermally robust up to ca. 500–600 °C and decomposes at still higher temperatures to the metal sulfides and sulfur [14]. Temperature levels around 500 °C are commonly used in the synthetic work-up of kesterite, also, to stabilize the structure of the most stable tetragonal polytype in the materials’ forms of rather thick layers or nanopowders [1,5,6,7,8,15,16]. 

Copper(I) sulfate (cuprous sulfate) Cu_2_SO_4_ is unstable under ambient air conditions and oxidizes easily to copper(II) sulfate CuSO_4_ [17]. The hydrated copper(II) sulfates have a general formula of CuSO_4_•nH_2_O, with *n* ranging from 1 to 7. The commonly encountered pentahydrate CuSO_4_•5H_2_O contains structurally inequivalent water molecules, and it dehydrates in three steps, as supported by a TG–DSC study employing an impressively wide range of heating rates [18]. The first two steps, taking place at 50–150 °C, consist of losing four water molecules in two consecutive events, each releasing two of them, whereas the final dehydration of the fifth water molecule is shown to occur at ca. 170–260 °C. In another similar study of this compound, the loss of four water molecules was found to occur by 90 °C and that of the last one was completed by 215 °C [19]. Regarding the stability of the anhydrous/dehydrated CuSO_4_, its thermal decomposition with increasing temperature may take place in two steps due to the transient formation of copper oxysulfate CuO•CuSO_4_, as shown schematically in Reaction 1.

2CuSO_4_ → CuO•CuSO_4_ + ↑SO_3_ → 2CuO + ↑SO_3_ (1)

The temperatures of copper sulfate decomposition, which was investigated by TGA/DTA in the atmosphere of air, are quoted in the range of 600–880 °C, with the highest values assigned to the decomposition of the postulated oxysulfate [20,21]. Actually, the thermal decomposition of CuSO_4_•5H_2_O has been detailed in so many studies that it serves as an example for a thermal analysis application [22].

The hydrated zinc sulfates ZnSO_4_•nH_2_O, *n* = 1 to 7, are commonly exemplified by the stable monohydrate ZnSO_4_•H_2_O [23,24]. The latter compound is found by TGA to dehydrate in one step by 250 °C, which is followed by the decomposition of ZnSO_4_, first, at 700–800 °C to oxysulfate ZnO•2ZnSO_4_ and, then, at 800–950 °C to ZnO with the release of gaseous SO_3_, as outlined in Reaction 2 [24].

3ZnSO_4_•H_2_O → 3ZnSO_4_ + ↑3H_2_O → ZnO•2ZnSO_4_ + ↑SO_3_ → 3ZnO + ↑2SO_3_ (2)

In another study of the stability of ZnSO_4_•nH_2_O, the dehydration of the intermediate monohydrate ZnSO_4_•H_2_O was found to be effective at 224 °C, the formation of zinc oxysulfate started at 590 °C, and the final decomposition to ZnO was detected at >900 °C [23]. The comparison of the results for the copper and zinc sulfates supports their similar robustness towards decomposition, both in the dehydration stage and for the complete chemical decomposition to the respective oxides via the transient oxysulfates. This was partly confirmed in the TGA study comparing several anhydrous metal sulfates, which pointed out a relatively lower decomposition temperature—by ca. 50 °C—for the copper sulfate, whereas the DTA profiles showed two stages, i.e., consistent with the formation of the transient copper oxysulfate, followed by its decomposition to CuO, and only one stage for the direct decomposition of zinc sulfate to ZnO [20].

The homovalent tin sulfates, Sn(II)SO_4_ [25] and Sn(IV)(SO_4_)_2_ [26], and heterovalent Sn(II)Sn(IV)(SO_4_)_3_ [26] are the rare examples of crystalline tin compounds in the system Sn-S-O. Especially, the Sn(IV)-containing sulfates require skillful preparations and handling because they are subject to easy hydrolysis and colloidal SnO_2_•nH_2_O and H_2_SO_4_ formation [27]. The TGA study, conducted under a nitrogen atmosphere, provided the beginning of the decomposition for the pure compounds Sn(II)SO_4_ at 430 °C (towards SnO_2_ and SO_2_—Reaction 3) and Sn(IV)(SO_4_)_2_ at 580 °C (towards SnO_2_ and SO_3_—Reaction 4) [27].

SnSO_4_ → SnO_2_ + ↑SO_2_ (3)

Sn(SO_4_)_2_ → SnO_2_ + ↑2SO_3_ (4)

The decomposition temperatures for both tin sulfates were found to be distinctly lower than those for the similar copper and zinc derivatives. In another thermogravimetric study on the stability of anhydrous Sn(SO_4_)_2_, the beginning of its decomposition under a nitrogen atmosphere was determined at ca. 420 °C, and the high hydrolytic susceptibility of the compound was confirmed with the formation of amorphous SnO_2_, after being exposed in ambient air as a thin layer for a few hours [28]. It is also worth acknowledging the recently increased interest in the so-called sulfated SnO_2_—the [SO_4_]^−2^-functionalyzed SnO_2_ that employs surface sulfate groups to act on such a support as a superacid catalyst in various organic syntheses [29]. The existence of the sulfated SnO_2_ derivatives is in line with the amphoteric characteristics of the oxide.

Yet another chemical reaction system can be involved when investigating kesterite nanopowders made by a “wet” mechanochemical synthesis route. The application of chemically inert, high-boiling-point organic liquids (e.g., alcohols, xylene, various hydrocarbons) for making a slurried reaction environment results at the end in the retention of some organics in the product nanopowders. The organic remnants may then decompose to carbonaceous species upon thermal annealing under neutral gas conditions. In this regard, the thermally treated kesterite nanopowders were recently shown by micro-Raman spectroscopy to contain some residual carbon [5]. The carbon contaminant is expected to react with such oxygen-bearing gases as O_2_, H_2_O, and SO_2_/SO_3_, which will impact the nature of the gaseous species that evolve upon heating the nanopowders. Specifically, there are numerous reactions possible in the C-O-S system and, for instance, the presence of CO, CO_2_, COS, CS_2_, SO_2_, SO_3_, O_2,_ and S_2_ in the reactions of carbon with sulfur oxides was predicted theoretically and confirmed in some relevant studies [30,31]. The examples of a few important reactions in the C-O-S system are shown below (Reactions 5–9). The course of these reactions, which are also involved in numerous equilibria, will depend, other things being equal, on temperature. However, the rising temperature at a freely selected heating rate, coupled with possible numerous heterogeneous reactions under gas flow conditions in the TGA/DTA-QMS experiments, make it very hard to even estimate the prevailing pathways.

2SO_3_ → 2SO_2_ + O_2_ (5)

2SO_2_ + 2C → 2CO_2_ + S_2_ (6)

CO_2_ + C → 2CO (7)

SO_2_ + 2C → CO + COS (8)

2COS → CO_2_ + CS_2_ (9)

In the current study, a pool of kesterite-type nanopowders was first prepared via the “wet” mechanochemical synthesis method from two precursor systems, i.e., from the mixture of constituent elements (2Cu + Zn + Sn + 4S) (labeled CE) [6] and from the in situ-made copper zinc/tin alloys (formed in the prior high-energy ball milling of the metals 2Cu + Zn + Sn) and sulfur (+4S) (labeled CA) [7]. The raw products containing xylene were conventionally dried overnight in air, stored in a desiccator, and annealed at 500 °C to make the final tetragonal polytype nanopowders. These materials were used in the already-published investigation of their prolonged oxidation in ambient air [5]. Herein, we approached the kesterite oxidation phenomena from a specific direction offered by the application of thermogravimetry and thermal analysis coupled with mass spectroscopy (TGA/DTA-QMS), the latter for the detection of evolving gas/volatile compounds at rising temperatures. First, the measurements under neutral argon gas provided data on the synthesis-related oxygen contents that originated in humid, ambient air conditions, as well as on the high-temperature compound’s stability up to 1000 °C. Second, the investigation under the flow of the oxygen-containing gas mixture of O_2_:Ar = 1:4 (vol.) delivered characteristics of the compound’s susceptibility to oxidation at elevated temperatures under the dry, oxidizing gas mixture. Both measurements provided with the complementary results related to the oxidation phenomena in the synthesis and storage of the quaternary sulfide Cu_2_ZnSnS_4_ nanopowders.

## 2. Experimental Section

### 2.1. Preparation of Kesterite Nanopowders

Two precursor systems were used to make the powders using mechanochemistry via high-energy ball milling (Pulverisette 7 model, Fritsch, Idar-Oberstein, Germany), as already described by us. The first system [6], labeled CE, was made of the (C)onstituent (E)lements in stoichiometric proportions, i.e., copper Cu, zinc Zn, tin Sn, and sulfur S (2Cu + Zn + Sn + 4S), with 2 at% excess of S, which were slurried with xylene and, subsequently, milled in the grinding bowl for 16 hrs at 1000 rpm. Upon xylene overnight drying/evaporation in air, the resulting free-flowing nanopowder of cubic prekesterite was subjected to the thermal treatment under the flow of argon at 500 °C for 6 hrs, yielding a black nanopowder of tetragonal kesterite. In the second system [7], labeled CA, the in situ-made (C)opper Zn/Sn (A)lloys from the prior high-energy ball milling of the metal powders (2Cu + Zn + Sn) for 10 hrs at 900 rpm were further milled with a 2 at% excess of sulfur vs. stoichiometry for 4 hrs at 900 rpm yielding upon xylene evaporation a prekesterite nanopowder. This material was annealed under an argon flow at 500 °C for 6 hrs to result in the tetragonal kesterite nanopowder. All final nanopowders after preparation were immediately stored in the desiccator, and their use in characterization and measurements was scrutinized towards a minimum exposure to air.

### 2.2. Characterization and TGA/DTA-QMS Measurements

Powder XRD determinations were carried out on the Empyrean PANalytical diffractometer (Malvern Pananalytical Ltd., Malvern, UK), Cu Kα source, 2Θ = 10–110°, and average crystallite sizes were estimated from Scherrer’s equation, applying the Rietveld refinement method. The oxygen contents in the starting metals and in the kesterite nanopowders were directly determined with the ONH836 elemental analyzer (Leco Corporation, St. Joseph, MI, USA) using multiple measurements on samples weighing 0.01–0.02 g. The analyzer uses the inert gas fusion technique for the simultaneous determination of the oxygen, nitrogen, and hydrogen contents in a range of inorganic materials. The TDA/DTA-QMS measurements were performed using the thermal analyzer STA 449 F3 Jupiter Netzsch (Netzsch Gerätebau GmbH, Selb, Germany) in the temperature range from ambient to 1000 °C with the heating rate of 10 °C/min, gas flow during the initial 30 min stabilization period of 15 mL/min, gas flow during measurements of 30 mL/min, gas flow during cooling period of 5 mL/min, and the sample mass of ca. 30 mg. The thermogravimetric and thermal analyses were performed for each nanopowder (i) under the neutral gas atmosphere of argon and (ii) under the oxidizing atmosphere of a mixture of oxygen and argon, O_2_:Ar = 1:4 by volume. The off-gas analysis at pre-selected m/e values was performed by the quadrupole mass spectrometer QMS 403C Aëolos, which was coupled to the TGA/DTA analyzer by a heated quartz capillary.

## 3. Results and Discussion

The use of two precursor systems in the mechanochemical synthesis of the kesterite-type nanopowders provided a pool of four products for the study, i.e., two raw cubic prekesterites and two annealed tetragonal kesterites. Given the specific details of the synthetic procedures and various starting oxygen contents in the substrates, while applying the same temperature level of annealing, the prepared nanopowders are chemically the same but synthetically diverse from the structure, morphology, and effectiveness of the oxidation points of view. The materials’ synthetic diversity should result in scattered oxygen contents due to adventitious oxidation on fortuitous exposure to ambient air, which could be then related to the structure/morphology characteristics. Such an approach is thought to provide more versatile and better-substantiated results compared, for instance, with an alternative case study conducted for a single kesterite nanopowder.

**Figure 1:**
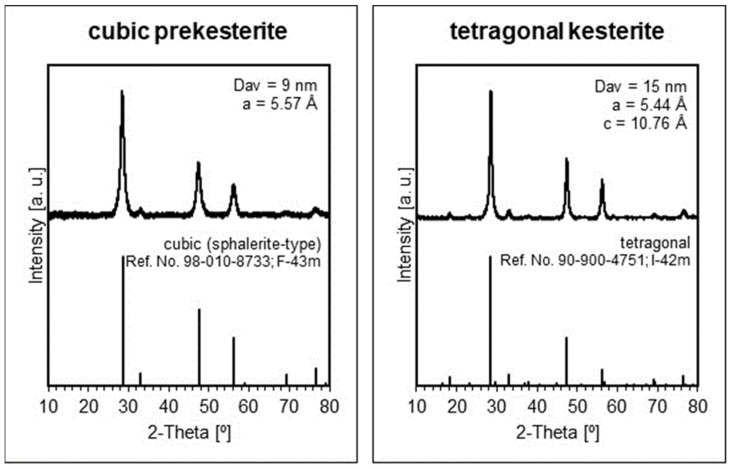
Top row—XRD patterns with lattice parameters *a*/*a*,*c* and average crystallite sizes *D_av_* for left—cubic (prekesterite) and right—tetragonal (kesterite) nanopowders prepared in the CA system. Bottom row—bar charts for cubic (sphalerite-type) and tetragonal polytypes.

The XRD patterns for the raw, as-made nanopowders from both systems are qualitatively identical and confirm the initial formation of the cubic prekesterite, while the respective patterns for the annealed nanopowders support the presence of tetragonal kesterite. Figure 1 illustrates the patterns for the materials from the copper alloy system CA, and they can be matched with the published relevant results in the constituent element system CE [5]. The calculated lattice parameters for the cubic prekesterite and tetragonal kesterite are very similar to those initially reported for the products in both systems [6,7]. And, similarly, the currently quoted respective average crystallite sizes of 9 nm and 15 nm are coherent with the literature values supporting the crystal growth that accompanies the thermal annealing stage and the formation of the stable tetragonal polytype. It is worth pointing out that, according to our experience in the area, the sensitivity of the powder XRD determinations is roughly of the order of 1 wt% and could be even worse for nanopowder components with crystallites in the low-nanosized regime. Consequently, small quantities of plausible crystalline oxidation by-products might not be detected by the method. This is the case for the hydrated copper and zinc sulfates that were confirmed in the freshly made nanopowders in small quantities by FT-IR spectroscopy but were not detected by XRD. However, a few months-long exposure of the kesterite nanopowders to ambient air confirmed substantial oxidation with the hydrated metal sulfates as the dominant by-products that were confirmed by XRD [5].

The presence of oxygen-bearing components in all the nanopowders is supported by the direct oxygen-content determinations [5]. These contents are reproduced in Table 1, with the additional oxygen-content data for the metal substrates. Worth noticing are the high oxygen levels in the 4–5 wt% range for the raw prekesterite nanopowders from both preparation systems and clearly lower amounts in the range of 0.6–1.6 wt% for the annealed kesterite nanopowders. They all can be compared with the values of ca. 0.1–0.6 wt% for the metal substrates. The outstanding high-oxygen contents for the prekesterites are consistent with an efficient post-synthesis oxidation likely taking place during xylene evaporation from the freshly made material slurries exposed for several hours to ambient air. The lower contents in the kesterites are probably due to thermal decomposition of some of the primary kesterite oxidation products, such as the hydrated metal sulfates with SO_3_/SO_2_ and H_2_O release at the annealing stage and, generally, due to lower oxidation reactivity of the recrystallized tetragonal polytype.

**Figure 2:**
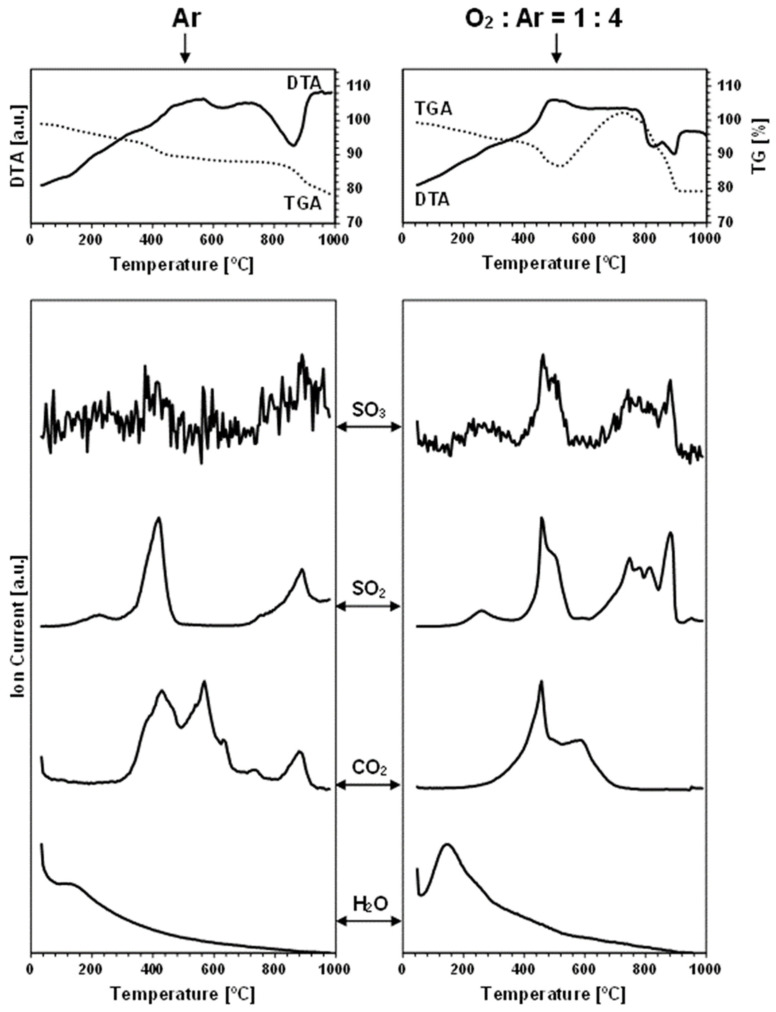
TGA/DTA-QMS determinations for prekesterite nanopowder from CE system. Left column—neutral Ar atmosphere, right column—oxidizing atmosphere of O_2_:Ar = 1:4 (vol.). The top row shows thermogravimetric (TGA—dotted line) and thermal (DTA—solid line) changes, whereas the rows below show gas evolution curves for m/e corresponding, from top to bottom, to SO_3_, SO_2_, CO_2_, and H_2_O.

**Figure 3:**
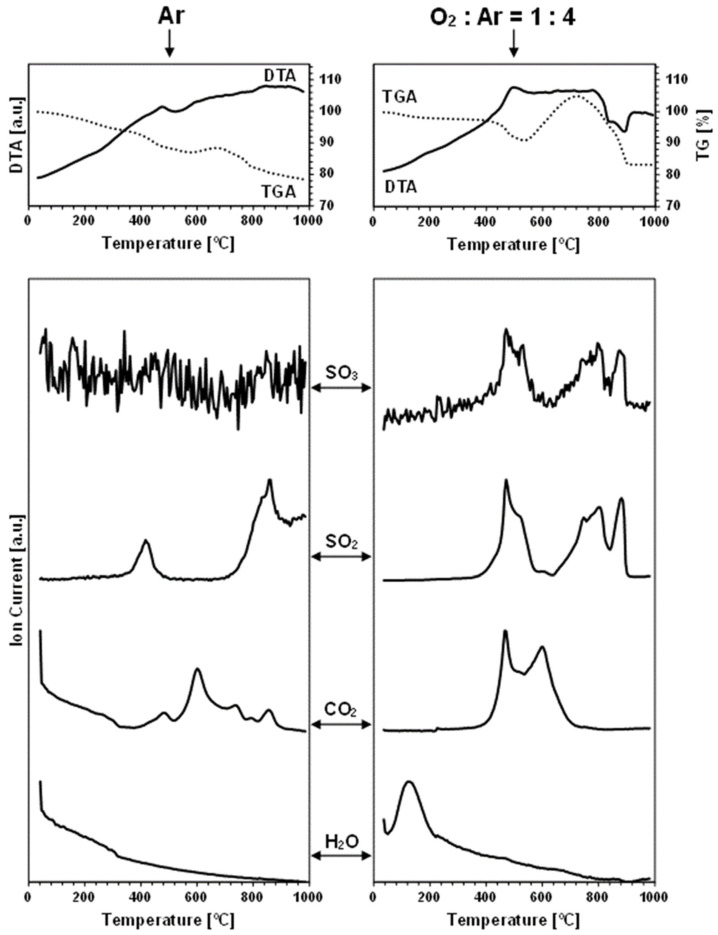
TGA/DTA-QMS determinations for kesterite nanopowder from CE system. Left column—neutral Ar atmosphere, right column—oxidizing atmosphere of O_2_:Ar = 1:4 (vol.). The top row shows thermogravimetric (TGA—dotted line) and thermal (DTA—solid line) changes, whereas the rows below show gas evolution curves for m/e corresponding, from top to bottom, to SO_3_, SO_2_, CO_2_, and H_2_O.

**Figure 4:**
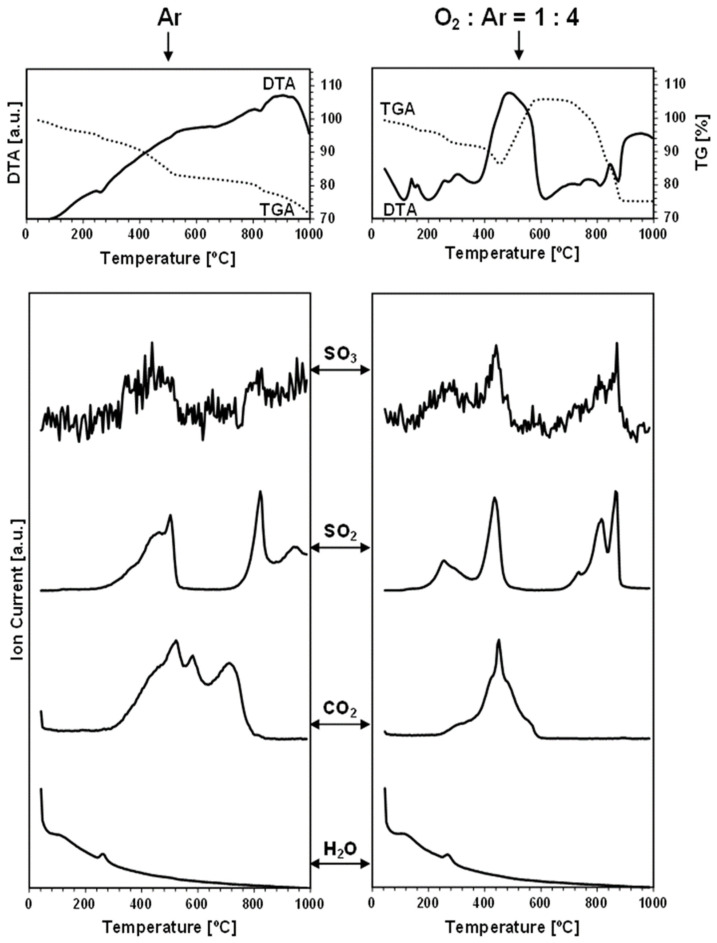
TGA/DTA-QMS determinations for prekesterite nanopowder from CA system. Left column—neutral Ar atmosphere, right column—oxidizing atmosphere of O_2_:Ar = 1:4 (vol.). The top row shows thermogravimetric (TGA—dotted line) and thermal (DTA—solid line) changes, whereas the rows below show gas evolution curves for m/e corresponding, from top to bottom, to SO_3_, SO_2_, CO_2_, and H_2_O.

**Figure 5:**
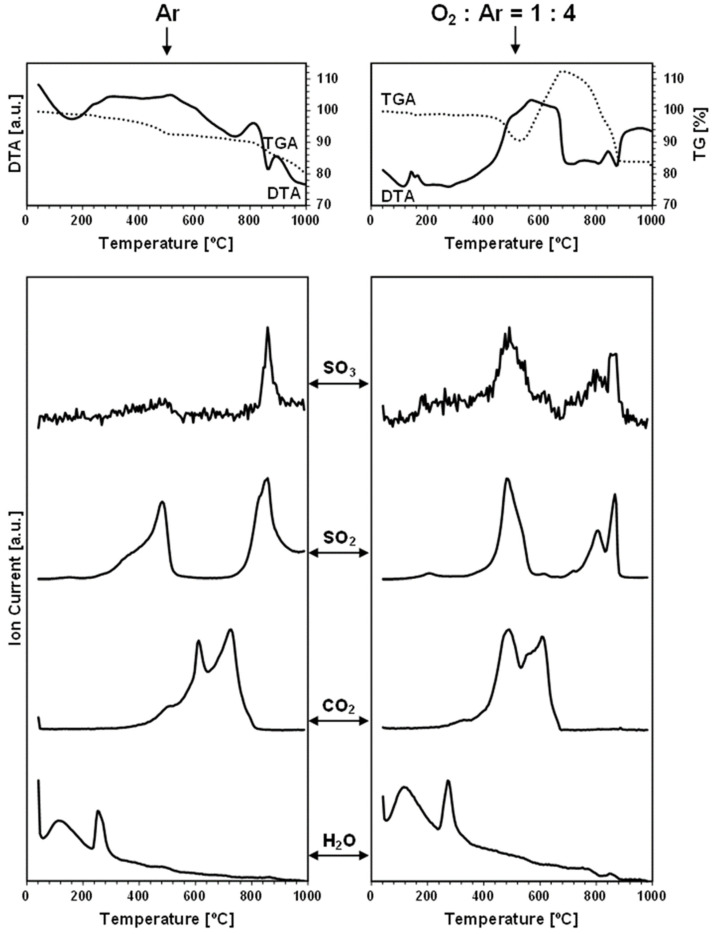
TGA/DTA-QMS determinations for kesterite nanopowder from CA system. Left column—neutral Ar atmosphere, right column—oxidizing atmosphere of O_2_:Ar = 1:4 (vol.). The top row shows thermogravimetric (TGA—dotted line) and thermal (DTA—solid line) changes, whereas the rows below show gas evolution curves for m/e corresponding, from top to bottom, to SO_3_, SO_2_, CO_2_, and H_2_O.

The TGA/DTA-QMS experiments were carried out under an argon gas flow and under an oxygen-containing O_2_:Ar = 1:4 (vol.) gas mixture in temperatures up to 1000 °C. The spectroscopic m/e analysis of the off-gases was set for the detection of such probable oxygen-containing gaseous molecules as O_2_, H_2_O, CO_2_, SO_2_, and SO_3_, but also for a few other plausible volatile species, like N_2_/CO, S_4_, S_8_, SnO, and SnS [32]. As noted, the neutral gas conditions were used to probe the evolution of oxygen-bearing compounds already present in the nanopowders, i.e., adventitiously formed in the conventional synthesis that was completed with the storage of the materials in the desiccator, as described in the Experimental Section. On the other hand, the measurements under the dry, oxygen-containing O_2_:Ar = 1:4 (vol.) gas mixture were primarily intended to evaluate the kesterite nanopowders’ susceptibility to forced oxidation at increased temperatures. Figure 2 and Figure 3 contain the results of the measurements for, respectively, prekesterite and kesterite from the CE system, each showing the data recorded for the two kinds of gas atmosphere. Figure 4 and Figure 5 include the same data for, respectively, prekesterite and kesterite from the CA system.

The thermogravimetric traces for all the nanopowders in the neutral gas atmosphere measurements are distinctly different from those recorded under the oxidizing conditions. Based on the recorded data, Table 2 includes the estimated weight changes in the characteristic temperature ranges under both gas atmospheres. The key differentiating feature is a pronounced weight increase beginning in the case of the oxidizing atmosphere above 460–540 °C, which will be discussed in detail later. 

In argon, the TG curves for the prekesterites and kesterites from both the CE and CA systems are characteristic of, roughly, three-step weight changes, i.e., first, a several wt% decrease up to ca. 500 °C; second, a relatively stable weight in the range of 500–800 °C with merely a 2–3 wt% drop; and, finally, a several wt% decrease in the range of 800–1000 °C. Regarding these ranges, the thermally annealed kesterite nanopowders show visibly smaller weight losses of the order of 2–7 wt% up to 500 °C compared with the related weight losses of 11–16 wt% for the raw prekesterite nanopowders. This is followed by the similar and small weight changes up to ca. 800 °C, and, further, by a more pronounced drop to 1000 °C. All this is consistent with a better thermal stability of the kesterites compared with the parent prekesterites, at least up to the temperatures in the 700–800 °C range, after which both forms clearly decompose, most likely to the metal sulfides and sulfur [14,32,33,34,35]. Also, the prekesterite from the CA system clearly shows a larger wt% loss up to 500 °C, as well as a total loss up to 1000 °C compared with its counterpart from the CE system, and this is also true for the related pair of kesterites when comparing the losses up to 500 °C. These pronounced differences underline the synthesis-linked variations in the properties of the otherwise chemically matched nanopowders. It is also instructive to note that the total weight losses up to 1000 °C under the neutral argon flow are generally higher compared to the oxidizing gas atmosphere. This is consistent with the formation of the stable and non-volatile metal oxides (CuO, ZnO, SnO_2_) upon oxidation compared with the prevailing formation of the metal sulfides from kesterite decomposition under argon with some of the latter (e.g., SnS_2_, SnS [14]), showing significant volatility in this temperature range.

In the measurements under argon, performed for the prekesterites and kesterites from both systems, the detected oxygen-bearing gaseous compounds are SO_3_, SO_2_, CO_2_, and H_2_O. At the same time, no other presumed volatile species, such as S_4_, S_8_, SnO, and SnS, are found. Both sulfur oxides first start to evolve in the range of 300–500 °C, with a maximum intensity close to 400–500 °C, then fall to a detection background level and, subsequently, rise from 700–750 °C to peak around 800–850 °C and, at times, also at 950 °C. It is worth underlining that the amounts of SO_3_ are much smaller than those of SO_2_, and that both evolution traces are well correlated with each other in the entire measurement range. This suggests that the primarily evolved sulfur compound is SO_3_, likely, from the decomposition of some sulfate groups present on the surface of the kesterite particles. Under the experimental conditions (suitably high temperatures, gas flow), the equilibration of Reaction 5 (vide intra) favors the increased proportions of SO_2_ and O_2_, as underlined in many metal sulfate decomposition studies [22,24,26,36]. In this regard, the evolution of the sulfur oxides taking place up to ca. 500 °C cannot be linked to the first step of decomposition of copper and zinc sulfates towards the oxysulfate intermediates. The previous thermogravimetric studies indicated the beginning of such decomposition at still higher temperatures in the range ca. 480–600 °C [20,21,23], although it was evidenced that, for instance, a carbon additive substantially lowered the temperatures [23]. Following this question, there is no piece of evidence for the formation of tin sulfates in kesterite oxidation, while the decomposition temperatures in separate studies are quoted for them at temperatures as low as 420–430 °C [27]. Regarding a postulated kesterite oxidation pathway in ambient air with a presumed transient Sn(SO_4_)_2_, the compound, if formed, is to undergo hydrolysis with the formation of pure crystalline or amorphous hydrated tin(IV) oxide, according to Reaction 10 [28].

Sn(SO_4_)_2_ + nH_2_O → SnO_2_•(n − 2)H_2_O + 2H_2_SO_4_ (10)

The adsorbed-on-kesterite-nanopowders sulfuric acid H_2_SO_4_ from this reaction could be a source of the evolved SO_3_/SO_2_ in the first temperature range below 500 °C since the non-catalytic thermal decomposition of H_2_SO_4_ to H_2_O and SO_3_ was reported to take place efficiently at 300–450 °C [37]. And, finally, the distinct second temperature range of the SO_3_/SO_2_ evolution above 700–750 °C is consistent with a complete desulfurization by progressing decomposition of the sulfates of copper and zinc (see, Reactions 1 and 2). Interestingly, the SO_3_/SO_2_ thermogravimetric peaks above 800 °C are accompanied by the exothermic DTA effects (Figure 2, Figure 3, Figure 4 and Figure 5), which supports facile oxidation of the metal sulfides originating from the thermally decomposed kesterite nanopowders [32]. Here, the most likely oxygen source for the exothermic oxidation would be the SO_3_ evolution, with the compound’s prevailing decomposition to SO_2_ and O_2_ taking place at the highest experimental temperatures (Reaction 5). As an illustration, the two distinct exothermic events at ca. 800 and 900 °C, seen clearly for the kesterite from the CA system (Figure 5), which are correlated with the SO_3_/SO_2_ evolution in this temperature range, may correspond with the consecutive decomposition of the copper and zinc sulfates towards the CuO and ZnO, respectively [36].

Each carbon dioxide CO_2_ evolution curve recorded under argon includes three to four events spread in the wide temperature range of 300 °C (Figure 2 and Figure 4)–400 °C (Figure 3 and Figure 5) to 800 °C (Figure 4 and Figure 5)–900 °C (Figure 2 and Figure 3). The presence of CO_2_ is proposed to be an artefact of using xylene solvent that was not efficiently removed/evaporated in the final mechanochemical synthesis step (see Experimental Section). Some of this organic compound is thought to remain adsorbed in the raw cubic prekesterite, whereas it is likely to be decomposed to carbon species during annealing in argon at 500 °C towards tetragonal kesterite. The compounds involved in forming CO_2_ are possibly the sulfur oxides, SO_3_, and/or SO_2_, which are also known to oxidize carbon towards CO, COS, and CS_2_ (see, Reactions 5–9). This notion is supported by the observation that the start of the CO_2_-evolution curves either coincides with the SO_3_/SO_2_ curves (true for the prekesterite samples, Figure 2 and Figure 4) and/or it is a bit delayed relative to the latter curves (true for the kesterite samples, Figure 3 and Figure 5). The prekesterites from both precursor systems appear to be more reactive towards carbon oxidation compared with the relevant kesterites since the carbon dioxide starts to evolve in the former cases at temperatures lower by ca. 100 °C. The oxidation of the remnant xylene in the prekesterite is apparently more facile than that of the pyrolyzed carbons in the kesterite. The origin of a few peaks in the CO_2_ evolution remains unknown. However, their appearance, which is spread across a range of a few hundred degrees, supports a multistep evolution of the carbon-oxidizing SO_3_/SO_2_ compounds. It is interesting to note that CO_2_ itself can also be, under specific conditions, an oxidizing medium for some metal sulfides and be reduced to CO [38,39]. In this regard, the potential CO evolution in the current mass spectroscopy measurements is entangled with the signal for N_2_, with these two molecules having the same m/e of 28. We were following the detection of m/e at 28, but in all cases, the curves were consistent with some adsorbed N_2_, the amounts of which quickly decayed with temperature to barely measurable levels after reaching the 200–300 °C range.

Finally, the water vapor H_2_O evolution curves confirm higher proportions of H_2_O in both nanopowders from the CA systems. In the nanopowders from both systems, the distinct H_2_O evolution peaks appear near 120 °C and in the neighborhood of 250–260 °C. The former peak is consistent with desorption of physically adsorbed water, although the detailed thermogravimetric studies of CuSO_4_•5H_2_O and ZnSO_4_•7H_2_O decomposition also support a release of a number of water molecules of crystallization at 50–150 °C [18,23]. The second H_2_O evolution peak fits the dehydration event for the last water molecule from CuSO_4_•H_2_O, found to occur at 173–260 °C [18], and from ZnSO_4_•H_2_O recorded at 224–252 °C [23]. It is appropriate to note that there are no clearly shaped H_2_O evolution peaks for the kesterite from the CE system, but, rather, enhanced levels of H_2_O evolution are seen up to ca. 300 °C (Figure 3). This can be confronted with the lowest oxygen content of 0.60% among the samples (Table 1) and, likely, resulted in very small quantities of sulfates in that sample. Overall, the evolution of water vapor is consistent with the presence of the hydrated metal sulfates and, therefore, supports some oxidation of both nanocrystalline kesterite polytypes on fortuitous and often indiscriminate exposure to ambient air.

**Figure 6:**
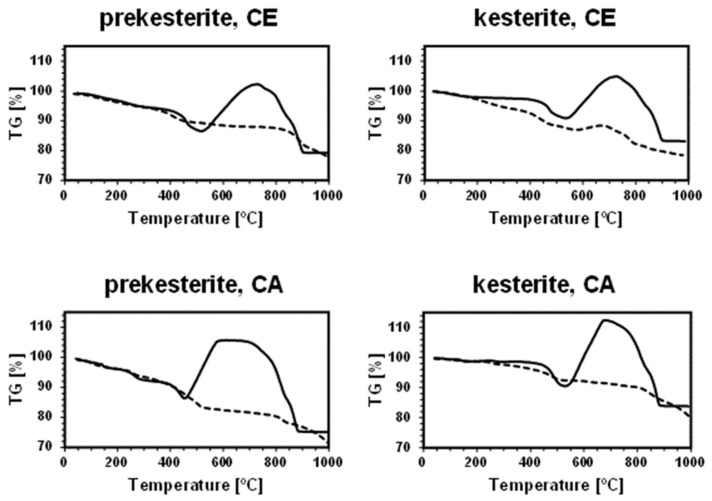
Paired thermogravimetric TG curves under neutral Ar atmosphere (dashed lines) and oxidizing atmosphere O_2_:Ar = 1:4 (solid lines) for: left column—nanopowders of prekesterite, right column—nanopowders of kesterite; from: top row—precursor system CE, bottom row—precursor system CA.

As mentioned before, the thermogravimetric curves under the oxidizing atmosphere of O_2_:Ar = 1:4 (vol.) show distinct differences compared with the neutral argon atmosphere, while, at the same time, they display remarkable similarities among themselves. Figure 6 illustrates this with the paired TG curves for the oxidizing and neutral gas measurements for the nanopowders from both systems. The curves under the oxidizing conditions are characteristic of the initial weight decrease to ca. 400 °C, which parallels quite well the decrease under the neutral argon flow, and which is followed by a clearly steeper decrease in the range of 400–500 °C and, then, by a pronounced weight increase taking place at ca. 460–(600)700 °C. From this stage, the attained maximum weight persists in the increasing temperature range of 50–150 °C, dropping, upon reaching 880–900 °C, to a constant weight that is stable to the final temperature of 1000 °C (Figure 2, Figure 3, Figure 4 and Figure 5, Table 2). The first stage up to 400 °C is thought to reflect the common changes for both atmospheres related to evaporation of the adsorbed species and dehydration of the hydrated sulfates, which do not depend greatly on the gas atmosphere. The steep weight decrease at 400–500 °C coincides with the concerted evolution of SO_3_/SO_2_ and CO_2_, the latter convincingly supporting an efficient carbon burnout with the now available ample oxygen. This is confirmed by a strong exothermic feature in the DTA curve in the range of 400–700 °C. The pronounced weight increase starting at ca. 460–540 °C is consistent with the beginning of kesterite oxidation, specifically, via the oxidation of the sulfide S^−2^ moieties towards the sulfate [SO_4_]^−2^ groups. This is also an exothermic reaction, as seen in the DTA curve in this temperature range. The beginning of the weight gain happens at a lower temperature for the more reactive prekesterite than for the related kesterite, as supported by the respective pairs of the temperatures for the system CE (520 and 540 °C) and system CA (460 and 530 °C). It is interesting to relate the total weight changes here to the theoretical values that are calculated based on the most likely kesterite oxidation to the stable oxides of (2CuO + ZnO + SnO_2_) or, less likely, of (Cu_2_O + ZnO + SnO_2_) [20]. In the former case, the anticipated weight decrease amounts to 11.0 wt%, whereas, in the latter, it is 14.6 wt%. The actual values are in the range of 11–21 wt% (Table 2), which is consistent with some additional kesterite oxidation weight decrease pathways likely existing below ca. 450–500 °C and, thus, before the major oxidation reactions begin, as supported by the data in Table 2. Some of the weight decrease could also be assigned to oxidation of the carbon impurity and its removal from the system as CO_2_ gas.

The evolution of SO_3_/SO_2_ under the oxidizing conditions also takes place in two temperature ranges, as previously discussed for the neutral gas conditions (Figure 2, Figure 3, Figure 4 and Figure 5). First, at the relatively low temperatures in the range of 400–550 °C, which is now raised by ca. 50–100 °C compared with the argon conditions, the presumed decomposition of sulfuric acid contributes mostly to the evolution of the sulfur oxides. And, at still higher temperatures, starting at ca. 650 °C for the system CE and at ca. 700 °C for the system CA, the beginning of decomposition of the metal sulfates formed by the prior oxidation of kesterite with the oxygen-containing gas is the likely source of SO_3_/SO_2_. Interestingly, that high temperature range is more complex now than under the neutral gas conditions. This could be looked at as the consequence of an efficient oxidation of kesterite towards the individual metal sulfates that decompose shortly afterwards with metal oxide formation via the oxysulfate derivatives at the definite temperatures below 880–900 °C [22,23,24,25,26]. The evolution of SO_3_/SO_2_ and its completion by 880–900 °C is the final decomposition event, and no further weight changes take place in any of the nanopowders up to the final temperature of 1000 °C.

The CO_2_ evolution curves recorded under the mixture of oxygen and argon consistently display two maxima at ca. 450–500 and 600 °C and almost no evolution at still higher temperatures, which are otherwise seen under argon. The first maximum coincides with the first SO_3_/SO_2_ evolution step at the lower temperatures, suggesting that the in statu nascendi oxygen, from decomposition of the evolving SO_3_, reacts after all with the available carbon. The second and final carbon oxidation event for all the nanopowders, at ca. 600 °C, is likely associated with reactions of oxygen from the oxygen-containing gas mixture with the remaining quantities of carbon. This step appears highly unspecific and ends up at ca. 700 °C with no further CO_2_ evolution, in accordance with the reported efficient oxidation in air of many nanocarbons by 600–800 °C [40,41]. The carbon-related oxidation is clearly exothermic, as confirmed by the DTA curve in this temperature range.

The water vapor presence in the off-gases analyzed under the oxidizing conditions follows the general trend described earlier for the argon gas flow. The H_2_O-related peaks are detected in two quite clearly defined temperature ranges, i.e., at 120–150 and 270–280 °C. Similar to the case of argon flow, these ranges are associated with the release of a few loosely bound water molecules of crystallization from the hydrated metal sulfates and with the more tightly bound molecules of this type given off at the higher temperatures, respectively. The lack of clearly evolved H_2_O peaks at ca. 270–280 °C while detecting the peaks at 120–150 °C for the nanopowders from the system CE (Figure 2 and Figure 3) can be understood both in terms of extremely low amounts of the sulfates in the kesterite (Table 1) and, possibly, the specific decomposition conditions of the prekesterite in this system. Nevertheless, the confirmed specific H_2_O evolution constitutes important evidence for a quite efficient partial oxidation of the kesterite-type nanopowders towards the hydrated metal sulfates already in the synthesis and storage/manipulation stages.

## 4. Conclusions

The susceptibility to two distinct oxidation processes of the range of kesterite nanopowders prepared by the mechanochemically assisted synthesis method is addressed by the TGA/DTA-QMS measurements under the neutral Ar gas and under the oxidizing O_2_:Ar = 1:4 (vol.) gas mixture conditions. On the one hand, the neutral gas conditions address, after all, the oxidation effects imparted onto the nanopowders along the synthesis pathway and the associated fortuitous materials’ exposure to ambient humid air. It is confirmed that the as-made nanopowders are significantly oxidized during this stage, mostly with the formation of the hydrated metal sulfates. The thermogravimetry, coupled with thermal and mass-spectroscopic analyses of the off-gases, confirm this finding by detecting the thermally driven evolution of SO_2_/SO_3_ and H_2_O from the nanopowders. Additionally, interesting aspects of the contaminant carbon oxidation by the evolving sulfur oxides towards CO_2_ are noted. Under the conditions of the measurements in argon, the kesterite nanopowders are found thermally stable up to 700–800 °C. On the other hand, the application of the dry oxidizing gas conditions provides data concerned with the action of oxygen on the kesterite nanopowders at increased temperatures up to 1000 °C. The characteristic sample weight increase stage, due to specific oxidation of the nanopowders of two kesterite polytypes, begins in the narrow temperature range of 460–540 °C and starts at slightly lower temperatures for the more reactive cubic polytypes. It is worth underlining that, under the TGA/DTA-QMS conditions, all the nanopowders appear quite robust in the dry, oxidizing O_2_:Ar = 1:4 gas mixture at the relatively high temperatures in a few-hundred-degrees range.

## Figures and Tables

**Table 1:** Directly determined oxygen contents for nanopowders of kesterite products and metal substrates in the CE and CA precursor preparation routes.

Oxygen Content [wt%]						
CE System		CA System		Metal Substrates		
Prekesterite	Kesterite	Prekesterite	Kesterite	Cu	Zn	Sn
4.48	0.63	4.32	1.60	0.59	0.08	0.17

**Table 2:** Weight changes [wt%] in characteristic temperature ranges derived from the thermogravimetric curves for prekesterite and kesterite nanopowders in the TGA/DTA-QMS experiments under argon and oxygen–argon gas atmospheres.

		Ar		O_2_:Ar = 1:4 (vol.)		
Nanopowder	Weigh Loss to 500 °C	Weight Loss at 500–800 °C	Total Weight Loss to 1000 °C	Weight Loss to 500 °C	Weight Gain at 460–700 °C	Total Weight Loss to 1000 °C
		[wt%]			[wt%]	
prekesterite, CE	11	2	22	13	16	21
kesterite, CE	2	2	20	9	14	17
prekesterite, CA	16	3	28	9 (<460 °C)	20	20
kesterite, CA	7	3	20	8 (4)	21	11

## Data Availability

Data are contained within the article.
